# Opportunities and Challenges in Democratizing Immunology Datasets

**DOI:** 10.3389/fimmu.2021.647536

**Published:** 2021-04-16

**Authors:** Sanchita Bhattacharya, Zicheng Hu, Atul J. Butte

**Affiliations:** ^1^ Bakar Computational Health Sciences Institute, University of California, San Francisco, San Francisco, CA, United States; ^2^ Department of Pediatrics, University of California, San Francisco, San Francisco, CA, United States

**Keywords:** immunology, open-access, democratization, data reuse, public repositories

## Abstract

The field of immunology is rapidly progressing toward a systems-level understanding of immunity to tackle complex infectious diseases, autoimmune conditions, cancer, and beyond. In the last couple of decades, advancements in data acquisition techniques have presented opportunities to explore untapped areas of immunological research. Broad initiatives are launched to disseminate the datasets siloed in the global, federated, or private repositories, facilitating interoperability across various research domains. Concurrently, the application of computational methods, such as network analysis, meta-analysis, and machine learning have propelled the field forward by providing insight into salient features that influence the immunological response, which was otherwise left unexplored. Here, we review the opportunities and challenges in democratizing datasets, repositories, and community-wide knowledge sharing tools. We present use cases for repurposing open-access immunology datasets with advanced machine learning applications and more.

## Introduction

Over the last decade, the field of immunology has exploded in an unprecedented way with exciting scientific breakthroughs, the rapid expansion of immunologic techniques, and the development of cutting-edge analytical tools (1–3). Studies have shown that complex diseases such as cancer, autoimmune disorders, and infections can be tackled by manipulating the immune system to fight against disease anomalies (4). The community is witnessing a plethora of data generated from high-throughput technically advanced experiments, large-scale clinical trials, multi-institution government-funded projects resulting in a data-rich environment.

In the 21st century, the democratization of domain-specific knowledge has become essential and vital to disrupt the silos created over many decades. There are several ongoing efforts to democratize datasets, circumvent gatekeepers and reduce bottlenecks to the data gateways. Large government funded projects are launched to encourage the research investigators for collaboration, conduct academic training, and workshops for promoting the field of data science (5, 6). Here, we discuss the opportunities and challenges posed to biomedical research in democratizing immunology datasets.

## Harnessing Large-Scale Immunology Datasets

There are numerous open science initiatives launched globally with the support and recognition from the research community in the last decade (7–9). As a result, there is an exponential growth in the number of repositories with a broad range of applications funded by government agencies, private and non-profit organizations. Scientific publishers and research funders are also releasing new data-sharing mandates to make the scientific findings transparent and reproducible. According to one registry of research data repositories (re3data.org), there are more than 2000 open, 1000 closed, and 350 embargoed research data repositories (10). Some repositories require users to submit data access proposals reviewed by independent data access committees, which can be a very time consuming and tedious process (11). This suggests that one needs to jump through many hoops to search and access public datasets in the existing systems.

Moreover, research has become more interdisciplinary than ever before, and scientists must broaden their search across disciplines or less familiar areas. With advances in technology and the availability of big data, there is a paradigm shift toward data-driven hypothesis to get novel biological insight (12). To advance scientific discoveries, the data management practices including data collection, ingestion, integrity, and governance following the Findability, Accessibility, Interoperability and Reusability (FAIR) principles are extremely necessary for responsible data sharing (13).

### Discoverability

There is a wide gap between articles in journals and associated data. The research community has an unmet need to store and share well-annotated large volumes of discrete experimental data to facilitate data reuse. For example, there has been a rapid expansion of flow cytometry applications in the last few years. However, only a handful of cytometry data deposition and sharing portals such as ImmPort (immport.org) (14) and Flowrespository.org (15) collect and share raw and/or processed data associated with experimental findings. A large portion of other immune measurements such as Enzyme-Linked Immunosorbent assay (ELISA), Hemagglutination Inhibition Assay (HAI), Luminex assays for cytokine profiling are primarily found embedded in supplementary files associated with the publication and are hard to discover.

In 2019, The Google Dataset Search (https://g.co/datasetsearch), a web-based dataset-discovery tool, was built using a crowdsourcing approach for sharing information about data repositories across a broad scope – social science, life science, physics, climate science, and beyond (16). The flexibility in sharing the datasets in flat files, tabular, or any other digital format based on indexing the metadata (data about data) makes it unique. The portal relies on an open ecosystem where dataset providers publish semantically enhanced metadata on their sites. The tool aggregates, normalizes, and reconciles metadata, providing a search engine that lets users find datasets on the web. Dataverse (dataverse.org) is a major international collaborative project led by Harvard’s Institute for Quantitative Social Science (IQSS), that facilitate public distribution of persistent, authorized, and verifiable data. Each dataset in the Dataverse contains descriptive metadata and data files (including documentation and analysis code that accompany the data). Dataverse has developed data citation standards that offers proper recognition to authors and permanent identification through global identifiers (17, 18).

### Accessibility

Data accessibility is one of the key drivers in accelerating reproducible science, increasing transparency, and repurposing the shared data to enhance scientific knowledge. In the last decade, the data sharing awareness through generalist and domain-specific repositories are exponentially growing and embraced by the community (19).

To facilitate accessibility, data sharing sites are developing Graphical User Interface (GUI), Applied Programming Interface (API) tools, and cloud-based resources to cater broad spectrum of users - experimentalists, clinicians, computational biologists, citizen scientists (5, 14). Furthermore, several projects have been launched that deliver harmonized immunology datasets around a specific theme using the Shiny web application (shiny.rstudio.com) with R (r-project.org). For example, we developed a curated immunology reference set of 10,000 Immunomes (10kimmunomes.ucsf.edu), which was synthetically built from a subset of healthy individuals, with no experimental manipulation. These datasets were harmonized and aggregated across many studies, and available for free download to the research community (20). Another data management and analysis resource, ImmuneSpace, leverages large-scale datasets, generated by the Human Immunology Project Consortium (HIPC) to characterize the immune system under normal conditions and in response to various stimuli (21, 22).

### Interoperability

The research field of systems immunology uses mathematical approaches and computational methods to examine the interactions between cellular and molecular networks within the immune system. One of the major barriers in integrating multi-scale immunology datasets from disparate sources is lack of annotation and metadata standardization, variation in analyte names, ambiguity in measurement units, data aggregation and more. For example, immunophenotyping experiments requires careful attention to reagents, sample handling, instrument setup, and data analysis, and is essential for successful cross-study and cross-center comparison of data (23). The HIPC data standards working group leveraged the ontologies to cross-compare cell types and marker(s) expression of each cell type referred as gating definitions in immunophenotyping. They crowdsourced large sets of gating definitions and corresponding cell types from ImmPort studies to examine the ability to parse gating definitions using terms from the Protein Ontology (PRO) and cell type descriptions from Cell Ontology (CL) (24). The Adaptive Immune Receptor Repertoire (AIRR) Community is developing a set of standards for describing, reporting, storing, and sharing adaptive immune receptor repertoire data, such as sequences of antibodies and T cell receptors (TCR) (25). As we move toward the use of machine learning and artificial intelligence, controlled vocabularies are critical. Even more critical is the need for robust definitions of the clinical phenotypes and diagnoses that accompany these samples to ensure the accurate comparison between cases and controls.

The crosstalk between the federated resources hosted by private, public, and government-funded agencies is minimal under the existing condition. For example, cancer researchers seeking clinical and omics data from other disease areas such as rheumatology have no easy solution to retrieve datasets. There is a lack of common data elements that would facilitate interoperability between two disease areas. The National Cancer Institute (NCI) Cancer Research Data Commons (CRDC) had started integrating datasets and analytical tools to share, integrate, analyze, and visualize cancer research data to enable interoperability between the NIH cloud resources and external resources (26). One such great example of interoperability was initiated between Cancer Genomics Cloud (CGC) and ImmPort powered by Seven Bridges (27). A pilot project was launched to host specialized rheumatology datasets from ImmPort within the CGC ecosystem and create opportunities for cancer researchers to integrate disease datasets beyond cancer.

## Benefits of Democratizing Immunology Resources

With the growing importance of open data for promoting reproducible science and building data ecosystems, the challenge is to conglomerate immunology related datasets and repositories to facilitate information exchange and ultimately facilitate broader adoption and democratization of datasets and tools by the biomedical research community.

### Democratization of Immunology Datasets

In the past few years, democratizing clinical research, trials, patient health record data is on the rise. There are long term benefits of minimizing the duplicative effort of building and supporting multiple independent database systems across institutions. Connecting data resources would drastically reduce the labor, time, and effort for the discoverability and accessibility of the datasets. Instead, funding can be effectively used to build the infrastructure to support interoperability.

Data commons and ecosystems are getting widely adopted for distributing biomedical data with cloud computing infrastructure and commonly used software services, tools, and applications for the large-scale management, analysis, harmonization, and sharing of biomedical data (28). For example, The NIH’s Big Data to Knowledge (BD2K) initiative established a virtual environment to facilitate interoperability and discoverability of shared digital objects accessible by a diverse community of researchers through the biomedical and healthCAre Data DIscovery Ecosystem (bioCADDIE) data discovery index commonly referred as DataMed (datamed.org) (17). ImmPort shares disparate immunology and clinical trials datasets spanning more than 30 National Institute of Allergy and Infectious Diseases (NIAID) programs and other external projects (14). ImmGen established by the Immunological Genome Project Consortium is a collaborative project between immunologists and computational biologists to understand the gene expression and regulatory networks in immune cells of the mouse (29). The iReceptor Scientific Gateway links distributed (federated) Adaptive Immune Receptor Repertoire (AIRR)-seq repositories provides access to a suite of tools for a complete analysis workflow, including modules for preprocessing and quality control of sequence reads, V(D)J gene segment assignment, repertoire characterization, and repertoire comparison (30).

With the recent outbreak of COVID-19 pandemic, data democratization and knowledge dissemination has become even more crucial. Large amounts of mechanistic and clinical immunology data are pouring in from the research and clinical community to understand the disease mechanism. For example, NIAID-funded multi-site Immunophenotyping Assessment in a COVID-19 Cohort (IMPACC) study is tracking and collecting the immunological measures from hospitalized patients to predict the clinical severity. The COVID-19 Prevention Network (COVPN) is a centralized clinical trial network established to test various vaccines and monoclonal antibodies as a preventive measure against COVID-19. There are ongoing efforts to build Human Cell Atlas, a comprehensive map of immune cells in health and disease (31).

### Democratization of Computational Applications

With increasing awareness for data sharing and dissemination, there is a rapid development of bioinformatics tools for harnessing such data. The day-to-day experience for many bench scientists, bioinformatic researchers, and tool developers involve generating new hypotheses, dealing with implementation details, overcoming technical barriers, and creating a distributed computing environment. The recent advances in cloud computing have democratized access to scalable and reproducible distributed systems for bioinformaticians and immunologists.

In 2015, the implementation of on-demand cloud-based storage and computing resources commonly known as Cloud Credits Model was developed by BD2K initiative which is now becoming popular in the biomedical research community. This model has three primary benefits: 1) provide access to datasets without having to download on the local machine 2) reduce economic and technological barriers to accessing and computing on large biomedical data sets *via* the STRIDES Initiative (8) cost and time efficient, as well as benefits such as speed, scalability, and interoperability from using cloud resources.

**Figure 1:**
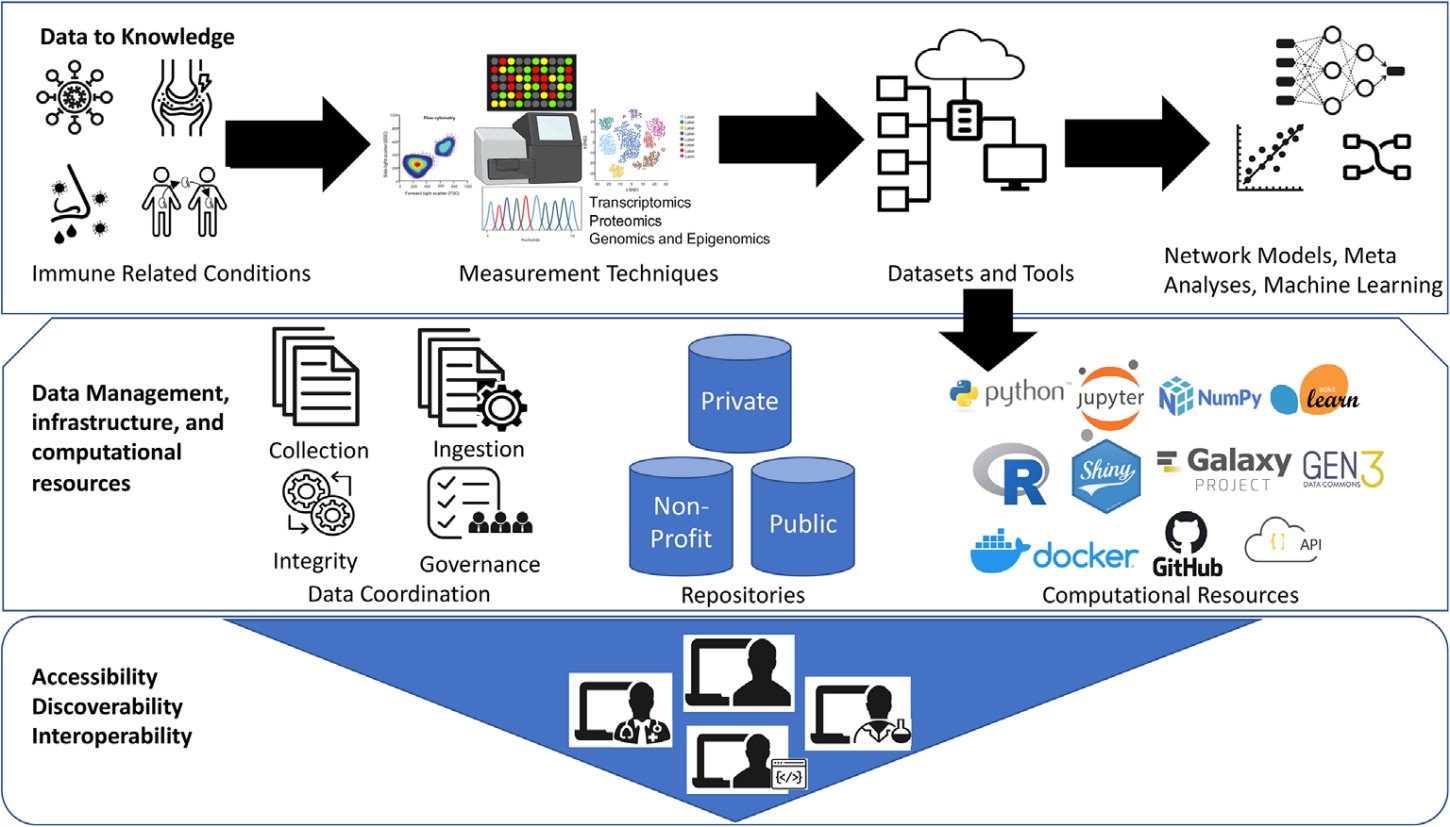
Democratization of datasets and computational tools. The Jupyter logo was used under Copyright © 2017 Project Jupyter Contributors. https://github.com/jupyter/jupyter.github.io/blob/master/assets/main-logo.svg; The Scikit learn logo is under Copyright © The scikit-learn developers. Source:-https://commons.wikimedia.org/wiki/File:Scikit_learn_logo_small.svg; NumPy logo source:- The NumPy logo is created by NumPy Team, 2020; https://github.com/numpy/numpy/blob/main/branding/logo/logomark/numpylogoicon.svg; Python logos are trademarks or registered trademarks of the Python Software Foundation, used with permission from the Foundation. Source:- https://legacy.python.org/community/logos/; Galaxy Project: https://galaxyproject.org/images/galaxy-logos/; Gen3:- The logo was used under the permission from Center for Translational Data Science at University of Chicago. Shiny- Shiny are trademarks of RStudio, PBC. https://github.com/rstudio/hex-stickers/blob/master/PNG/shiny.png; The R logo is © 2016 The R Foundation. (CC-BY-SA 4.0); Docker- Docker and the Docker logo are trademarks of Docker, Inc. in the United States and/or other countries. https://www.docker.com/company/newsroom/media-resources; Github- GITHUB®, the GITHUB® logo design are exclusive trademarks registered in the United States by GitHub, Inc, source:-https://github.com/logos.

The open-source bioinformatics software platform has been a great success over the years (32). One such great example is Bioconductor project (bioconductor.org), an open-source, open-development software project hosting wide-range of bioinformatic and statistical applications used for the analysis of high-throughput biological data, spanning from single-cell genomics to cytometry, and the list is rapidly growing (33). This distributed framework facilitated large-scale data integration and meta-analyses projects to promote secondary use of public datasets such as recount2 resource for RNA-Seq analysis (34) and other immunology datasets (35, 36). In addition, well cited bioinformatic analyses pipelines hosted on Galaxy (galaxyproject.org), GenePattern (genepattern.org), and other independent resources also provide flexibility to democratize bioinformatics tools. However, computational reproducibility and sharing analysis code with the published immunology studies is still lacking. The advent of software source code distribution and version control systems such as GitHub (github.com) and Docker Software (docker.com) which deploys all software dependencies required to run computational pipelines are some of the best practices that allows other people to more easily reproduce the analysis results. See **Figure 1**.

## Use Cases: Reuse of Shared Immunological Datasets

The number of available immunological datasets is growing faster than ever before, providing an unprecedented opportunity for researchers to repurpose data and generate new hypotheses. In this section, we highlight a few studies that leveraged publicly available datasets to address immunological questions. See **Table 1**.

**Table 1:** List of publications leveraging open-access immunological datasets.

Authors	Pubmed ID	Datasets	Study type	Description
Orange et al. (37)	29468833	Transcriptomics and histology	Machine learning	Identify RA subgroups using machine learning models
Hu et al. (38)	32801215	CyTOF	Machine learning	Identify latent CMV infection using a deep learning model
Gielis et al. (39)	31849987	TCR sequencing	Machine learning	Predict antigen specificity using a machine learning model
Berry et al. (40)	20725040	Transcriptomics	Meta-analysis	Identify transcription signature specific to active tuberculosis
Sweeney et al. (41)	27384347	Transcriptomics	Meta-analysis	Classify viral and bacterial infections using transcription signature
Jiang et al. (42)	30127393	Transcriptomics	Meta-analysis	Identify T cell suppression and exclusion signatures.
McClain et al. (43)	32743603	Transcriptomics	Biomarker analyses and validation using public datasets	Host response to SARS-CoV-2 infection through RNA sequencing
Kidd et al. (44)	26619012	Transcriptomics	Drug repurposing	Mapping the effects of drugs on the state-transition of immune cells

### Machine Learning Applications

Immune-profiling data are highly complex, with high-dimensionality and diverse sample types. Machine learning techniques are well suited to analyze complex immunological data. Multiple studies have demonstrated the potentials of machine-learning models to predict clinical related information (37, 45). Researchers have also leveraged various methods to interpret the machine-learning model and identified key immunological components (e.g., cytokines or cell subsets) that are associated with the clinical outcome of interest (38).

Orange et al. used supervised and unsupervised machine learning techniques to identify rheumatoid arthritis subtypes from the datasets generated by the Accelerating Medicines Partnership RA/SLE program, a public-private initiative of NIH. The study first used unsupervised clustering to identify three subtypes of rheumatoid arthritis from RNA-sequencing data. The researchers then trained a support vector machine (SVM) to predict the rheumatoid arthritis subtypes using histology features. The machine-learning algorithm allows doctors to classify rheumatoid arthritis into clinically relevant subtypes (37).

Hu et al. developed a deep learning model to analyze cytometry data. Using a convolutional neural network model, the deep learning model was able to take the raw cytometry matrices as input to predict clinical outcomes of interest. The study demonstrated that the deep learning model is able to accurately diagnose asymptomatic cytomegalovirus infection using Mass cytometry (CyTOF) data from the peripheral blood. In addition, the study developed a procedure to interpret the deep learning model. The procedure identified a subset of CD8+ T cells (CD27- CD94+ CD8+ CD3+) as a biomarker of latent cytomegalovirus infection (38). The deep learning model can also potentially be applied to diagnose other immune-related diseases, such as leukemia and autoimmunity.

Gielis et al. developed machine learning models to predict antigen specificity of TCR. The study utilized a massive amount of antigen-specific TCR sequences from immune repertoire databases, including McPAS-TCR and TCRdb. The study built a random forest-based machine learning model to identify TCR clones specific to a group of well-characterized antigens (39). The application allows researchers to identify disease or conditions that affect the antigen-specific T cells of known antigens.

### Meta-Analysis of Open-Access Immunology Datasets

Computational immunologists have also combined datasets from multiple studies to address scientific questions. A meta-analysis of existing data across different studies offers multiple benefits. The aggregated data allow researchers to test hypotheses with increased statistical power. The involvement of multiple independent studies increases the robustness of conclusions drawn. In addition, the complexity of aggregated data allows researchers to test or generate new hypotheses.

Berry et al. performed a cross-platform analysis of transcriptome data and identified transcript signatures to classify patients with active and latent tuberculosis, and later compared active tuberculosis with other inflammatory and infectious diseases. In addition, the study performed modular and pathway analysis and revealed that the tuberculosis disease signatures were dominated by interferon-induced gene expression change (40).

Sweeney et al. performed a meta-analysis to identify a transcriptional signature that can classify bacterial and viral-induced sepsis from eight public datasets containing 426 patient samples (142 viral and 284 bacterial infections). By comparing the viral and bacterial infections, the study identified a seven-gene signature that can classify viral and bacterial-induced sepsis. The signature was validated in 30 independent cohorts (41).

Jiang et al. leveraged large tumor cohorts from The Cancer Genome Atlas to identify signatures of T cell dysfunction that can predict cancer immunotherapy response (27). The study used Cox proportional hazards models to identify signatures of T cell dysfunction by testing how the expression of each gene in tumors interacts with the CTL infiltration level to influence patient survival. The signature predicted the outcome of melanoma patients treated with cancer immunotherapy. In addition, the approach was able to identify novel molecular targets to improve cancer immunotherapy, including SERPINB9, a granzyme B inhibitor (42).

During the COVID-19 pandemic, the scientific community has come together and started to share COVID-19 related datasets in the public domain, allowing other researchers to get additional insight into the datasets. For example, McClain et al. studied the transcript profiling differences between COVID-19 subjects and individuals with similar respiratory illnesses such as seasonal coronavirus, influenza, bacterial pneumonia, and matched healthy controls (43). The RNAseq analysis from peripheral blood mononuclear cells (PBMCs) revealed a distinctive interferon response, as well as the activation of coagulation and JAK/STAT signaling pathways, unique to COVID-19 patients. The study also derived two signatures that can distinguish COVID-19 patients from other respiratory infections and differentiate COVID-19 patients with mild and severe symptoms. The authors further validated the signatures using an independent datasets that is publicly available at Gene Expression Omnibus (GEO) (46, 47).

### Computational Drug Repurposing

The immune system plays critical roles in a variety of diseases. Modulating immune cells has been a common strategy for treating immune-related conditions. The shared immunological data has also been used to identify drugs that can modulate the immune system. Kidd et al. leveraged the datasets from the Library of Integrated Network-based Cellular Signatures (LINCS) project (48) and the ImmGen project to systematically characterize the interaction between drugs and immune cells (29). The study matched the drug-induced transcriptional signature with the signature of immune cell state transitions. The approach predicted 69,995 known and novel interactions. The study further validated the top predictions using electronic health record data and mouse models (44).

## Future Perspective

The field of Immunology is burgeoning with plenteous opportunities to understand the multicellular immune system at aggregate and single-cell resolution. To facilitate scientific discoveries without duplicative efforts, data democratization is a crucial step combined with the infrastructure and tools that support sharing and integration across multiple sources. One of the major goals of data accessibility is to allow a rich stream of data flow freely from source systems to researchers. To promote open-access data usage, data exploration tutorials, hands-on-workshops, and application programming training would better prepare future scientists. This should begin from ground level by introducing a data science course from high school to graduate curriculum across all the disciplines. To take advantage of the big data in immunology harbored in generalist or domain-specific repositories and ecosystems, streamlining data access, leveraging cloud-based resources, commonly used bioinformatics tools by the immunologists and researchers across various domains would help to scale the datasets and its usage globally.

## Author Contributions

SB formulated the original idea, and AB reviewed and approved the manuscript. SB contributed to the design of the review. SB and ZH wrote and reviewed the manuscript and designed the table and figure. All authors contributed to the article and approved the submitted version.

## Funding 

This work was supported by the National Institute of Allergy and Infectious Diseases ImmPort contract HHSN316201200036W. The content is solely the responsibility of the authors and does not necessarily represent the official views of the National Institutes of Health.

## Conflict of Interest

AB is a co-founder and consultant to Personalis and NuMedii; consultant to Samsung, Mango Tree Corporation, and in the recent past, 10x Genomics, Helix, Pathway Genomics, and Verinata (Illumina); has served on paid advisory panels or boards for Geisinger Health, Regenstrief Institute, Gerson Lehman Group, AlphaSights, Covance, Novartis, Genentech, and Merck, and Roche; is a shareholder in Personalis and NuMedii; is a minor shareholder in Apple, Facebook, Google, Microsoft, Sarepta, 10x Genomics, Amazon, Biogen, CVS, Illumina, Snap, Nuna Health, Assay Depot, Vet24seven, Regeneron, Moderna, and Sutro, and several other non-health related companies and mutual funds; and has received honoraria and travel reimbursement for invited talks from Genentech, Takeda, Varian, Roche, Pfizer, Merck, Lilly, Mars, Siemens, Optum, Abbott, Celgene, AstraZeneca, AbbVie, Johnson and Johnson, Westat, and many academic institutions, state or national agencies, medical or disease specific foundations and associations, and health systems. AB receives royalty payments through Stanford University, for several patents and other disclosures licensed to NuMedii and Personalis. AB’s research has been funded by NIH, Robert Wood Johnson Foundation, Northrop Grumman (as the prime on an NIH contract), Genentech, Johnson and Johnson, FDA, the Leon Lowenstein Foundation, the Intervalien Foundation, Priscilla Chan and Mark Zuckerberg, the Barbara and Gerson Bakar Foundation, and in the recent past, the March of Dimes, Juvenile Diabetes Research Foundation, California Governor’s Office of Planning and Research, California Institute for Regenerative Medicine, L’Oreal, and Progenity. SB and ZH are funded by ImmPort (under UCSF sub-contract with Northrop Grumman).

## References

[1] Bendall SC Simonds EF Qiu P Amir ED Krutzik PO Finck R. Single-cell mass cytometry of differential immune and drug responses across a human hematopoietic continuum. Science (2011) 332:687–96. 10.1126/science.1198704

[2] Newell EW Sigal N Bendall SC Nolan GP Davis MM. Cytometry by time-of-flight shows combinatorial cytokine expression and virus-specific cell niches within a continuum of CD8+ T cell phenotypes. Immunity (2012) 36:142–52. 10.1016/j.immuni.2012.01.002 PMC3752833 22265676

[3] Cohen L Fiore-Gartland A Randolph AG Panoskaltsis-Mortari A Wong S-S Ralston J. A Modular Cytokine Analysis Method Reveals Novel Associations With Clinical Phenotypes and Identifies Sets of Co-signaling Cytokines Across Influenza Natural Infection Cohorts and Healthy Controls. Front Immunol (2019) 10:1338. 10.3389/fimmu.2019.01338 31275311 PMC6594355

[4] Demaria O Cornen S Daëron M Morel Y Medzhitov R Vivier E. Harnessing innate immunity in cancer therapy. Nature (2019) 574:45–56. 10.1038/s41586-019-1593-5 31578484

[5] Margolis R Derr L Dunn M Huerta M Larkin J Sheehan J. The National Institutes of Health’s Big Data to Knowledge (BD2K) initiative: capitalizing on biomedical big data. J Am Med Inform Assoc (2014) 21:957–8. 10.1136/amiajnl-2014-002974 PMC4215061 25008006

[6] Van Horn JD Fierro L Kamdar J Gordon J Stewart C Bhattrai A. Democratizing data science through data science training. Pac Symp Biocomput (2018) 23:292–303. 29218890 PMC5731238

[7] FAIRsharing Registry WG. connecting (meta)data standards, repositories and policies. RDA (2014). Available at: https://www.rd-alliance.org/group/fairsharing-registry-connecting-data-policies-standards-databases.html (Accessed February 17, 2021).

[8] STRIDES Initiative | Data Science at NIH. Available at: https://datascience.nih.gov/strides (Accessed February 17, 2021).

[9] Open-Access Data and Computational Resources to Address COVID-19 | Data Science at NIH. Available at: https://datascience.nih.gov/covid-19-open-access-resources (Accessed February 17, 2021).

[10] Vierkant P Pampel H Ulrich R Scholze F Kindling M Witt M. re3data - Open infrastructure for Open Science. Available at: https://gfzpublic.gfz-potsdam.de/pubman/faces/ViewItemOverviewPage.jsp?itemId=item_4323890 (Accessed October 6, 2020).

[11] Geifman N Bollyky J Bhattacharya S Butte AJ. Opening clinical trial data: are the voluntary data-sharing portals enough? BMC Med (2015) 13:280. 10.1186/s12916-015-0525-y 26560699 PMC4642633

[12] Bui AAT Van Horn JD NIH. BD2K Centers Consortium. Envisioning the future of “big data” biomedicine. J BioMed Inform (2017) 69:115–7. 10.1016/j.jbi.2017.03.017 PMC5613673 28366789

[13] Wilkinson MD Dumontier M Aalbersberg IJJ Appleton G Axton M Baak A. The FAIR Guiding Principles for scientific data management and stewardship. Sci Data (2016) 3:160018. 10.1038/sdata.2016.18 26978244 PMC4792175

[14] Bhattacharya S Dunn P Thomas CG Smith B Schaefer H Chen J. ImmPort, toward repurposing of open access immunological assay data for translational and clinical research. Sci Data (2018) 5:180015. 10.1038/sdata.2018.15 29485622 PMC5827693

[15] Spidlen J Breuer K Rosenberg C Kotecha N Brinkman RR. FlowRepository: A resource of 389 annotated flow cytometry datasets associated with peer-reviewed publications. Cytometry Part 390 A (2012) 81A:727–31. 10.1002/cyto.a.22106 22887982

[16] Brickley D Burgess M Noy N. , in: Google Dataset Search: Building a search engine for datasets in an open Web ecosystem. in The World Wide Web Conference WWW ‘19, New York, NY, USA: Association for Computing Machinery (ACM) (2019). pp. 1365–75. 10.1145/3308558.3313685

[17] King G. An Introduction to the Dataverse Network as an Infrastructure for Data Sharing. Sociological Methods Res (2007) 36:173–99. 10.1177/0049124107306660

[18] Trisovic A Durbin P Schlatter T Durand G Barbosa S Brooke D. Advancing Computational Reproducibility in the Dataverse Data Repository Platform. P-RECS ‘20: Proc 3rd Int Workshop Pract Reproducible Eval Comput Syst. (2020) 15–20. 10.1145/3391800.3398173

[19] Burns NS Miller PW. Learning What We Didn’t Know — The SPRINT Data Analysis Challenge. New Engl J Med (2017) 376:2205–7. 10.1056/NEJMp1705323 28445656

[20] Zalocusky KA Kan MJ Hu Z Dunn P Thomson E Wiser J. The 10,000 Immunomes Project: Building a Resource for Human Immunology. Cell Rep (2018) 25:513–22.e3. 10.1016/j.celrep.2018.09.021 30304689 PMC6263160

[21] Computational resources for high-dimensional immune analysis from the Human Immunology Project Consortium | Nature Biotechnology. Available at: https://www.nature.com/articles/nbt.2777 (Accessed November 24, 2020). 10.1038/nbt.2777 PMC4294529 24441472

[22] Sauteraud R Dashevskiy L Finak G Gottardo R. ImmuneSpace: Enabling integrative modeling of human immunological data. J Immunol (2016) 196:65–124.

[23] Finak G Langweiler M Jaimes M Malek M Taghiyar J Korin Y. Standardizing Flow Cytometry Immunophenotyping Analysis from the Human ImmunoPhenotyping Consortium. Sci Rep (2016) 6:20686. 10.1038/srep20686 26861911 PMC4748244

[24] Maecker HT McCoy JP Nussenblatt R. Standardizing immunophenotyping for the Human Immunology Project. Nat Rev Immunol (2012) 12:191–200. 10.1038/nri3158 22343568 PMC3409649

[25] Rubelt F Busse CE Bukhari SAC Bürckert J-P Mariotti-Ferrandiz E Cowell LG. Adaptive Immune Receptor Repertoire Community recommendations for sharing immune-repertoire sequencing data. Nat Immunol (2017) 18:1274–8. 10.1038/ni.3873 PMC5790180 29144493

[26] Grossman RL Heath AP Ferretti V Varmus HE Lowy DR Kibbe WA. Toward a Shared Vision for Cancer Genomic Data. N Engl J Med (2016) 375:1109–12. 10.1056/NEJMp1607591 PMC6309165 27653561

[27] Lau JW Lehnert E Sethi A Malhotra R Kaushik G Onder Z. The Cancer Genomics Cloud: Collaborative, Reproducible, and Democratized—A New Paradigm in Large-Scale Computational Research. Cancer Res (2017) 77:e3–6. 10.1158/0008-5472.CAN-17-0387 PMC5832960 29092927

[28] Welcome to Gen3. Available at: http://gen3.org/ [Accessed March 16, 2021]

[29] ImmGen at 15 | Nature Immunology . Available at: https://www.nature.com/articles/s41590-020-0687-4 (Accessed November 24, 2020). 10.1038/s41590-020-0687-4 32577013

[30] Breden F Luning Prak ET Peters B Rubelt F Schramm CA Busse CE. Reproducibility and Reuse of Adaptive Immune Receptor Repertoire Data. Front Immunol (2017) 8:1418. 10.3389/fimmu.2017.01418 29163494 PMC5671925

[31] Building a high-quality Human Cell Atlas | Nature Biotechnology . Available at: https://www.nature.com/articles/s41587-020-00812-4 (Accessed February 19, 2021). 10.1038/s41587-020-00812-4 33500565

[32] Huber W Carey VJ Gentleman R Anders S Carlson M Carvalho BS. Orchestrating high-throughput genomic analysis with Bioconductor. Nat Methods (2015) 12:115–21. 10.1038/nmeth.3252 PMC4509590 25633503

[33] Amezquita RA Lun ATL Becht E Carey VJ Carpp LN Geistlinger L. Orchestrating single-cell analysis with Bioconductor. Nat Methods (2020) 17:137–45. 10.1038/s41592-019-0654-x PMC7358058 31792435

[34] recount workflow: accessing over 70,000 human RNA-seq samples with Bioconductor. Available at: https://bioconductor.org/packages/release/workflows/vignettes/recountWorkflow/inst/doc/recount-workflow.html (Accessed February 22, 2021). 10.12688/f1000research.12223.1 PMC5621122 29043067

[35] Haynes WA Vallania F Liu C Bongen E Tomczak A Andres-Terrè M. Empowering Multi-Cohort Gene Expression Analysis to Increase Reproducibility. Pac Symp Biocomput (2016) 22:144–53. 10.1142/9789813207813_0015 PMC5167529 27896970

[36] Hu Z Jujjavarapu C Hughey JJ Andorf S Lee H-C Gherardini PF. MetaCyto: A Tool for Automated Meta-analysis of Mass and Flow Cytometry Data. Cell Rep (2018) 24:1377–88. 10.1016/j.celrep.2018.07.003 PMC6583920 30067990

[37] Orange DE Agius P DiCarlo EF Robine N Geiger H Szymonifka J. Identification of Three Rheumatoid Arthritis Disease Subtypes by Machine Learning Integration of Synovial Histologic Features and RNA Sequencing Data. Arthritis Rheumatol (2018) 70:690–701. 10.1002/art.40428 29468833 PMC6336443

[38] Hu Z Tang A Singh J Bhattacharya S. Butte AJ. A robust and interpretable end-to-end deep learning model for cytometry data. Proc Natl Acad Sci USA (2020) 117:21373–80. 10.1073/pnas.2003026117 PMC7474669 32801215

[39] Gielis S Moris P Bittremieux W De Neuter N Ogunjimi B Laukens K. Detection of Enriched T Cell Epitope Specificity in Full T Cell Receptor Sequence Repertoires. Front Immunol (2019) 10:2820. 10.3389/fimmu.2019.02820 31849987 PMC6896208

[40] Berry MPR Graham CM McNab FW Xu Z Bloch SAA Oni T. An interferon-inducible neutrophil-driven blood transcriptional signature in human tuberculosis. Nature (2010) 466:973–7. 10.1038/nature09247 PMC3492754 20725040

[41] Sweeney TE Wong HR Khatri P. Robust classification of bacterial and viral infections via integrated host gene expression diagnostics. Sci Transl Med (2016) 8:346ra91. 10.1126/scitranslmed.aaf7165 PMC5348917 27384347

[42] Jiang B Sun Q Tong Y Wang Y Ma H Xia X. An immune-related gene signature predicts prognosis of gastric cancer. Med (Baltimore) (2019) 98:e16273. 10.1097/MD.0000000000016273 PMC6635287 31277152

[43] McClain MT Constantine FJ Henao R Liu Y Tsalik EL Burke TW. Dysregulated transcriptional responses to SARS-CoV-2 in the periphery. Nat Commun (2021) 12:1079. 10.1038/s41467-021-21289-y 33597532 PMC7889643

[44] Kidd BA Wroblewska A Boland MR Agudo J Merad M Tatonetti NP. Mapping the effects of drugs on the immune system. Nat Biotechnol (2016) 34:47–54. 10.1038/nbt.3367 26619012 PMC4706827

[45] Plasma Proteomics Identify Biomarkers and Pathogenesis of COVID-19: Immunity. Available at: https://www.cell.com/immunity/fulltext/S1074-7613(20)30449-0?_returnURL=https%3A%2F%2Flinkinghub.elsevier.com%2Fretrieve%2Fpii%2FS1074761320304490%3Fshowall%3Dtrue (Accessed February 23, 2021). 10.1016/j.immuni.2020.10.008 PMC7574896 33128875

[46] Wilk AJ Rustagi A Zhao NQ Roque J Martínez-Colón GJ McKechnie JL. A single-cell atlas of the peripheral immune response in patients with severe COVID-19. Nat Med (2020) 26:1070–6. 10.1038/s41591-020-0944-y PMC7382903 32514174

[47] Barrett T Wilhite SE Ledoux P Evangelista C Kim IF Tomashevsky M. NCBI GEO: archive for functional genomics data sets—update. Nucleic Acids Res (2013) 41:D991–5. 10.1093/nar/gks1193 PMC3531084 23193258

[48] Subramanian A Narayan R Corsello SM Peck DD Natoli TE Lu X. A Next Generation Connectivity Map: L1000 Platform and the First 1,000,000 Profiles. Cell (2017) 171:1437–52.e17. 10.1016/j.cell.2017.10.049 29195078 PMC5990023

