# Supplementary material for: Opportunities and Challenges in Democratizing Immunology Datasets
Source: Front Immunol. 2021 Apr 16;12:647536. doi: 10.3389/fimmu.2021.647536 (PMC8086961; doi:10.3389/fimmu.2021.647536)
Supplement: Supplementary file 1 [file Table_1.docx]

Supplementary Note

Logo and Graphics Credits for Figure 1

| **Logo and Graphics** | **License** | **Download Link** |
| --- | --- | --- |
| 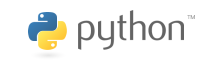 | 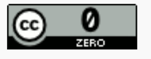"Python" is a registered trademark of the Python Software Foundation (PSF). The Python logos (in several variants) are use trademarks of the PSF as well. | https://www.python.org/community/logos/ |
| 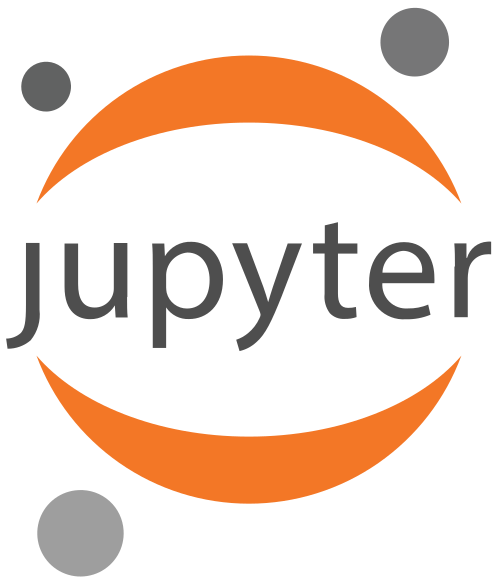 | Copyright © 2017 Project Jupyter Contributors  Creator: Cameron Oelsen | <https://github.com/jupyter/jupyter.github.io/blob/master/assets/main-logo.svg> |
| 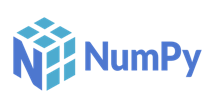 | 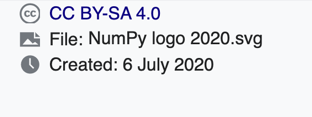Creator: Isabela Presedo-Floyd | <https://github.com/numpy/numpy/blob/main/branding/logo/logomark/numpylogoicon.svg> |
| 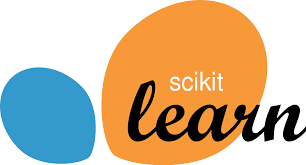 | 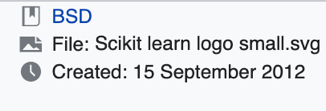Copyright © The scikit-learn developers | <https://github.com/scikit-learn/scikit-learn/blob/master/doc/logos/scikit-learn-logo.svg> |
| 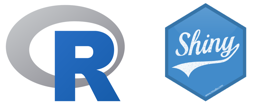 | The R logo is © 2016 The R Foundation.  CC-BY-SA 4.0 | https://www.r-project.org/logo/ |
| 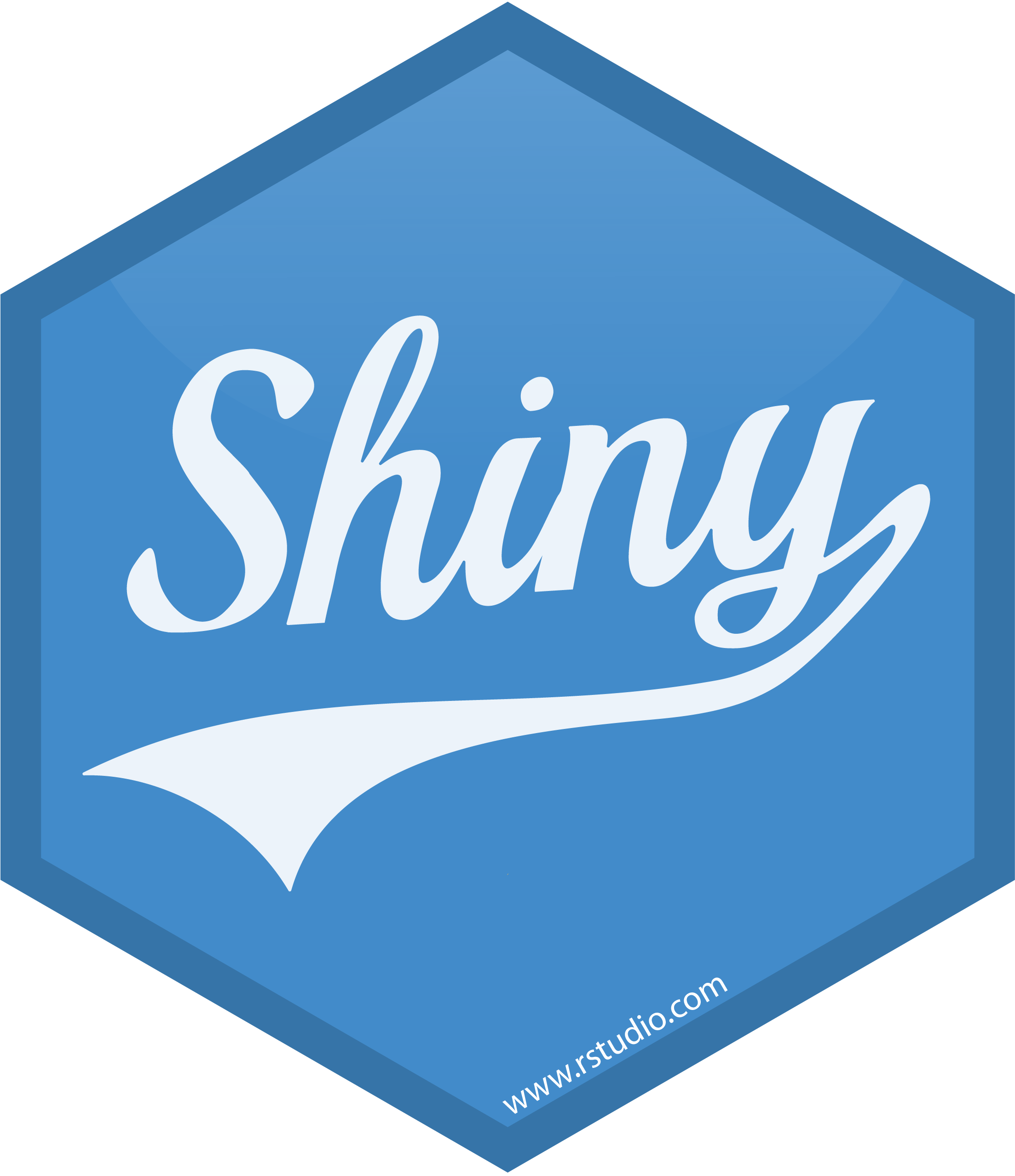 | RStudio and Shiny are trademarks of RStudio, PBC. | https://rstudio.com/about/trademark/) |
| 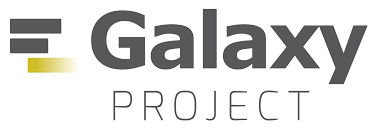 | Credit: The current version of the Galaxy Logo created by [Petr Kadlec](http://puradesign.cz/en). | [https://galaxyproject.org/images/galaxy-logos/galaxy_project_logo.png](https://galaxyproject.org/images/galaxy-logos/) |
| 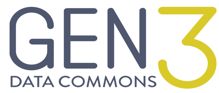 | Courtesy: Center for Translational Data Science @ University of Chicago. | https://ctds.uchicago.edu/gen3 |
| 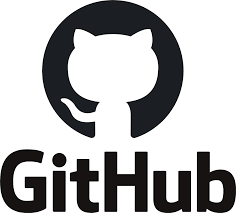 | GITHUB®, the GITHUB® logo design are exclusive trademarks registered in the United States by GitHub, Inc. | https://github.com/logos |
| 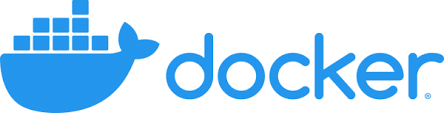 | Docker and the Docker logo are trademarks or registered trademarks of Docker, Inc. in the United States and/or other countries. Docker, Inc. and other parties may also have trademark rights in other terms used herein. | https://www.docker.com/company/newsroom/media-resources |
|   Measurement Techniques | The graphics for “Measurement Techniques” were created with BioRender.com. | Biorender.com |
